# Performance of ChatGPT, Gemini and DeepSeek for non-critical triage support using real-world conversations in emergency department

**DOI:** 10.1186/s12873-025-01337-2

**Published:** 2025-09-01

**Authors:** Sukyo Lee, Sumin Jung, Jong-Hak Park, Hanjin Cho, Sungwoo Moon, Sejoong Ahn

**Affiliations:** 1 Department of Emergency Medicine, Korea University Ansan Hospital, Ansan-si, 15355 Republic of Korea; 2 Core Research & Development Center, Korea University Ansan Hospital, Ansan-si, 15355 Republic of Korea; 3 Emergency Department, Korea University Ansan Hospital, 123, Jeokgeum-ro, Danwon-gu, Ansan-si, Gyeonggi-do Republic of Korea

**Keywords:** Triage, Artificial intelligence, Large language model, Clinical conversation, Korean triage and acuity scale

## Abstract

**Background:**

Timely and accurate triage is crucial for the emergency department (ED) care. Recently, there has been growing interest in applying large language models (LLMs) to support triage decision-making. However, most existing studies have evaluated these models using simulated scenarios rather than real-world clinical cases. Therefore, we evaluated the performance of multiple commercial LLMs for non-critical triage support in ED using real-world clinical conversations.

**Methods:**

We retrospectively analyzed real-world triage conversations prospectively collected from three tertiary hospitals in South Korea. Multiple commercial LLMs—including OpenAI GPT-4o, GPT-4.1, O3, Google Gemini 2.0 flash, Gemini 2.5 flash, Gemini 2.5 pro, DeepSeek V3, and DeepSeek R1—were evaluated for the accuracy in triaging patient urgency based solely on unsummarized dialogue. The Korean Triage and Acuity Scale (KTAS) assigned by triage nurses was used as the gold standard for evaluating the LLM classifications. Model performance was assessed under both a zero-shot prompting condition and a few-shot prompting condition that included representative examples.

**Results:**

A total of 1,057 triage cases were included in the analysis. Among the models, Gemini 2.5 flash achieved the highest accuracy (73.8%), specificity (88.9%), and PPV (94.0%). Gemini 2.5 pro demonstrated the highest sensitivity (90.9%) and F1-score (82.4%), though with lower specificity (23.3%). GPT-4.1 also showed balanced high accuracy (70.6%) and sensitivity (81.3%) with practical response times (1.79s). Performance varied widely between models and even between different versions from the same vendor. With few-shot prompting, most models showed further improvements in accuracy and F1-score.

**Conclusions:**

LLMs can accurately triage ED patient urgency using real-world clinical conversations. Several models demonstrated both high sensitivity and acceptable response times, supporting the feasibility of LLM in non-critical triage support tools in diverse clinical environments. These findings apply to non-critical patients (KTAS 3–5), and further research should address integration with objective clinical data and real-time workflow.

**Supplementary Information:**

The online version contains supplementary material available at 10.1186/s12873-025-01337-2.

## Background

Accurate and timely triage is essential for patient safety and optimal use of limited healthcare resources in the emergency department (ED) [1, 2]. Triage tools, such as the Emergency Severity Index (ESI) and the Canadian Triage and Acuity Scale (CTAS), are widely known to promote objectivity in triage decisions [3, 4]. In South Korea, the Korean Triage Acuity Scale (KTAS) was introduced nationally in 2016, adapted from the CTAS to better reflect local clinical environment [5]. This structured approach has improved consistency, patient flow, and overall quality management [6, 7]. 

The KTAS classifies patients into five levels of acuity. Levels 1 and 2 consist of patients with critical signs, such as altered mental status, shock, or respiratory failure, who are immediately prioritized for life-saving interventions and do not undergo routine conversational triage. In contrast, Levels 4 and 5 generally correspond to less urgent presentations. KTAS 4 patients may require re-evaluation within 1–2 h, while KTAS 5 patients typically represent non-urgent cases managed through observation or outpatient care. Although distinct in definition, both KTAS 4 and 5 categories are usually managed without urgent allocation of emergency resources. However, KTAS level 3 patients are not in need of immediate emergency intervention but may deteriorate and require medical management. Under-triage can compromise patient safety and resource allocation. Distinguishing Level 3 from Levels 4 and 5 is clinically challenging, as it depends more on nuanced patient interviews than on obvious critical signs. Therefore, accurate identification of KTAS 3 patients within the KTAS 3–5 group is crucial.

Over the past few years, large language models (LLMs)—including ChatGPT, Gemini, and, most recently, DeepSeek—have evolved rapidly. These models have evolved from simple text generation to more sophisticated abilities. Their capabilities now include advanced reasoning and step-by-step problem solving, enabled by chain-of-thought prompting [8]. The introduction of specialized reasoning models, designed to mimic human-like analytical thinking, has further expanded the range of tasks LLMs can address [9, 10]. 

These models are being adopted in medical settings for tasks ranging from clinical documentation to decision support. There is growing interest in leveraging LLMs for triage in the ED [11–15]. Early studies using clinical vignettes have reported that LLMs demonstrate acceptable performance in triage classification [11–14]. A recent prospective study found that ChatGPT could reach high agreement with clinicians in real-world triage using chief complaints, comorbidities, and vital signs [15]. 

However, most previous studies has been limited to vignettes or scenario-based data or has required various clinical data [11–15]. There is limited evidence regarding the performance of LLMs when using only real-world clinical conversations. In this study, we evaluated several state-of-the-art LLMs for their ability to classify patient urgency using unsummarized triage conversations between nurses and patients from EDs. This study specifically focused on the KTAS 3–5 patient group, with the primary aim of assessing whether LLMs can reliably identify KTAS 3 patients from real-world clinical conversations.

## Method

### Study design and setting

This was a retrospective observational study utilizing the Emergency Department Clinical Conversation Database. This database comprised prospectively collected, anonymized audio recordings and corresponding transcripts of interactions from three tertiary academic hospitals: Korea University Anam Hospital, Korea University Guro Hospital and Korea University Ansan Hospital. Data were collected from July to September 2022, overseen by the National Information Society Agency of Korea. The dataset was created to support the development of voice-based artificial intelligence (AI) applications in healthcare [16]. 

The dataset encompasses anonymized audio recordings and corresponding transcriptions from interactions between ED patients and healthcare providers. Clinical interactions in the database were categorized into four phases: initial triage assessment, physician consultation, diagnostic and medication procedures, and discharge instructions or test results discussion. Data collection was supervised by the Information Society Agency of Republic of Korea. Ethical clearance was obtained from the Institutional Review Boards (IRBs) of participating hospitals (IRB numbers: 2022AN0288, 2022GR0156, 2022AS0132).

Recording began with the initial interaction between provider and patient. Informed consent was obtained from the patient after triage, and recording continued throughout the rest of the ED encounter. If the patient did not consent to be recorded, all previous recordings were discarded. Patients categorized under KTAS levels 3, 4, and 5—representing urgent, semi-urgent, and non-urgent cases respectively—were included. Patients classified as KTAS 1 or 2, indicating resuscitation or immediate emergency treatment, were excluded to avoid any delay in urgent medical interventions caused by the consent process. Transcription and validation processes were conducted independently by two specialized transcription entities, ensuring accuracy and anonymity.

For this study, we only used conversation data from the triage phase. The goal was to assess the performance of various commercially available LLMs from OpenAI, Google, and DeepSeek in accurately classifying patient urgency based exclusively on these real-time, unsummarized clinical conversations.

### Ground truth validation

To validate the reliability of nurse-assigned triage levels, we performed an additional ground truth assessment. A total of 50 cases were randomly sampled from the dataset, preserving the original proportional distribution of KTAS levels 3, 4, and 5. Two board-certified emergency medicine physicians, each with over 10 years of clinical experience, independently reviewed these cases while blinded to both the nurse-assigned KTAS levels and the model predictions. In instances where their assessments differed, a third board-certified emergency physician with more than 10 years of experience adjudicated the case to establish a consensus label. The final consensus ratings were then compared with the original nurse triage levels, and agreement was evaluated using both the overall concordance rate and Cohen’s kappa coefficient (κ) to account for chance agreement.

### Large Language models and configuration

We evaluated the triage accuracy of multiple commercially available LLMs, including OpenAI GPT-4o, GPT-4.1, O3, Google Gemini 2.0 flash, Gemini 2.5 flash, Gemini 2.5 pro, and DeepSeek V3, DeepSeek R1. Of these, OpenAI O3, Google Gemini 2.5 pro, and DeepSeek R1 are known for their chain-of-thought prompting reasoning models. Specific versions and update dates are provided in Supplementary Table 1.

Two prompting strategies were employed. The first was a zero-shot prompt, which instructed the model only to classify triage levels according to the KTAS, serving as a baseline. The prompt instructed models to classify patient urgency exclusively as KTAS level 3 (urgent), 4, or 5 (not urgent), explicitly excluding KTAS levels 1 and 2, as these categories were not included in the dataset. The exact prompt is provided in Supplementary Table 2. The second was a few-shot prompt designed to provide additional guidance in situations where the available information was incomplete. In this approach, the model was instructed to infer severity from the conversational context when essential data such as vital signs were not explicitly available. Furthermore, in cases where uncertainty still remained, the model was encouraged to classify patients in a manner that minimized the risk of under-triage. To further support this reasoning process, the few-shot prompt also included one illustrative example for each of the three non-critical KTAS levels (3, 4, and 5). The complete prompt texts are presented in Supplementary Table 3. The few-shot example for KTAS level 3 is shown in Table 1, while the examples for KTAS levels 4 and 5 are provided in Supplementary Tables 4 and 5, respectively.

**Table 1:** An example of a clinical conversation in the emergency department used in the study (KTAS level 3). The original conversation is in korean, and the examples have been translated into english for better Understanding

No.	Speaker	Conversations
1	Triage nurse	This way please. Take a sit. What makes you come here today?
2	Patient	There was blood in my stool.
3	Triage nurse	Since when?
4	Patient	Yesterday.
5	Triage Nurse	What time was it yesterday?
6	Patient	7 o’clock.
7	Triage Nurse	7 p.m.?
8	Patient	No. In the morning.
9	Triage Nurse	This is the first time you’ve seen blood in your stool?
10	Patient	Yes.
11	Triage Nurse	Was it just blood in the stool or was the whole toilet red?
12	Patient	The whole toilet was red.
13	Triage Nurse	Did you only see one bloody stool?
14	Patient	No. Since yesterday.
15	Patient	Every time I went to the bathroom this evening and this morning.
16	Triage Nurse	The stool was red in all three times?
17	Patient	Yes.
18	Triage Nurse	Are you taking any medications?
19	Triage Nurse	Do you have hypertension or diabetes?
20	Patient	Both.
21	Triage Nurse	What else?
22	Patient	I take medication for thyroid.
23	Patient	I had thyroid cancer.
24	Triage Nurse	Are you feeling dizzy or anything?
25	Patient	Not really.
26	Triage Nurse	You’re not dizzy. Are you feeling out of breath?
27	Patient	No, not really.
…	…	…

All models were executed with a temperature of 0.0 to ensure deterministic outputs. The only exception was OpenAI’s O3 model, which is constrained to a fixed temperature of 1.0. Because OpenAI O3 operates with a fixed generation parameter (temperature = 1.0), it was excluded from the main performance comparison. However, its results are reported separately in the Supplementary Material. All other hyperparameters remained at default settings provided by the respective APIs.

### Data processing and input format

Original data were provided in JSON format and converted into natural conversation sequences without additional modification. Information outside of the conversation (e.g., vital signs) was not included in the analysis, but if it was included in the conversation, it was included. An example of a conversation is shown in Table 1. The original data were in Korean and used without translation.

### Outcome measurement and analysis

The model was instructed to output a single number classification corresponding to KTAS level 3, 4, or 5. Then, we binarized them into ‘urgent’ (KTAS 3) and ‘not urgent (KTAS 4 or 5). This binarized result was used to conduct statistical analysis. The classification performed by ED triage nurses was used as gold standard for comparison. Response times were measured by sequentially sending requests for all 1,057 cases and averaging the total time per request.

### Statistical analysis

To evaluate differences in triage classification performance among the LLMs, we utilized Cochran’s Q test. Post-hoc pairwise comparisons were conducted using the paired McNemar test, with Bonferroni correction. We calculated performance metrics, including accuracy, sensitivity, specificity, positive predictive value (PPV), negative predictive value (NPV), and F1-score. Confidence intervals (CIs) for sensitivity, specificity, and accuracy were calculated using exact Clopper-Pearson methods. Confidence intervals for predictive values were calculated using the standard logit confidence intervals method described by Mercaldo et al. [17]

### Failure analysis

To further explore the limitations of the proposed approach, we conducted a failure analysis based on the zero-shot prompt condition. We first identified the three models that achieved the highest overall accuracy and F1 scores. From these models, we extracted cases in which all three produced the same triage classification result, but this result did not match the nurse-assigned KTAS level. These discordant cases were then qualitatively reviewed to identify common characteristics or recurring patterns. The purpose of this analysis was to examine whether there are specific scenarios in which LLMs consistently fail, thereby revealing potential structural weaknesses shared across different systems. We selected the zero-shot condition for this analysis because it reflects the baseline performance of the models without additional guidance.

### Experimental environment

Analysis was conducted using Python (version 3.11.6) scripts executed through the PyCharm (version 2025.1.1.1) development environment. All models were accessed and tested through their respective official APIs. OpenAI and DeepSeek models were accessed via the Python OpenAI library, and Google models via the Google Generative AI (GenAI) Python library. Performance metrics and 95% confidence intervals were analyzed using MedCalc software (version 23.2.7; MedCalc Software Ltd., Ostend, Belgium).

### Ethical statement

This study was approved by the Institutional Review Board of Korea University Ansan Hospital (IRB No. 2025AS0116) and conducted in accordance with the principles of the Declaration of Helsinki. The requirement for informed consent was waived due to the retrospective analysis of publicly available anonymized data.

## Results

### Patient characteristics

A total of 1,057 patient triage conversations were analyzed to assess the triage performance of various commercially available LLMs. The baseline characteristics of these patients are detailed in Table 2. Of the total, 761 patients (72.0%) were classified as urgent (KTAS 3), and 296 patients (28.0%) were classified as non-urgent (KTAS 4 or 5). Among urgent patients, medical conditions accounted for 642 cases (84.4%), and injuries accounted for 119 cases (15.6%). Conversely, among non-urgent patients, medical conditions accounted for 143 cases (48.3%), while injuries accounted for 153 cases (51.7%). Three hundred sixty patients (47.3%) were men in urgent group and of the non-urgent patients, one hundred thirty-six patients (45.9%) were men in the non-urgent group. Age distribution indicated a higher proportion of patients aged 60 years or older in the urgent group, totaling 377 cases (49.5%), compared to 103 cases (34.8%) in the non-urgent group.

**Table 2:** Baselines characteristics of patients included in the study

Variables	Urgent (KTAS 3) *n* = 761	Not Urgent (KTAS 4 / 5) *n* = 296	Total *n* = 1,057
**Type of visit**			
Medical diseases	642	143	785
Injury	119	153	272
**Sex**			
Male	360	136	496
Female	401	160	561
**Age**			
20–29	76	60	136
30–39	85	42	127
40–49	87	41	128
50–59	136	50	186
60–69	182	55	237
≥ 70	195	48	243

KTAS, Korean Triage and Acuity Scale

### Validation of nurse-assigned triage levels

Among the 50 cases randomly sampled for validation, the distribution of nurse-assigned triage levels was 36 for KTAS 3, 11 for KTAS 4, and 3 for KTAS 5, consistent with the proportions observed in the overall dataset. When these assignments were compared with the consensus ratings from board-certified emergency physicians, 46 of the 50 cases (92.0%) were concordant. The agreement between nurse and physician labels corresponded to a Cohen’s kappa coefficient of 0.793 (95% CI, 0.601–0.985), indicating substantial agreement beyond chance. The four discordant cases all represented instances of under-triage by nurses, in which the consensus physician review assigned a higher KTAS level than the nurse assessment.

### Model performance with zero-shot prompting

**Fig. 1:**
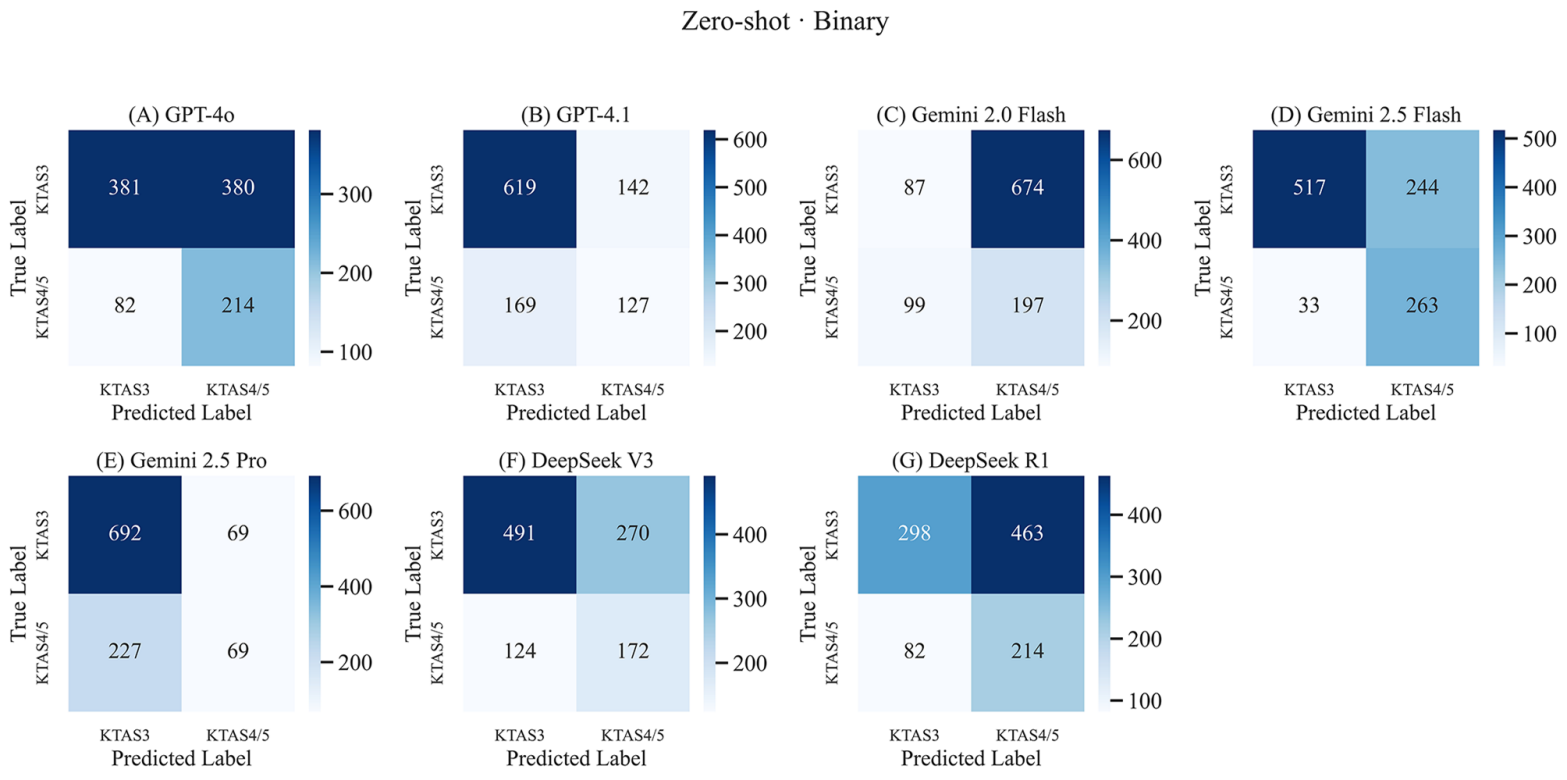
Binary confusion matrices for all evaluated models under the Zero-Shot condition. (**A**) GPT-4o (B) GPT-4.1 (**C**) Gemini 2.0 Flash (**D**) Gemini 2.5 Flash (**E**) Gemini 2.5 Pro (**F**) DeepSeek V3 (**G**) DeepSeek R1. Each heatmap illustrates classification outcomes for urgent (KTAS 3) vs. non-urgent (KTAS 4–5) patients

Table 3 summarizes the performance metrics of the evaluated LLMs under the zero-shot prompting condition in terms of accuracy, sensitivity, specificity, PPV, NPV, F1-score, and mean response time per case. Among the models in the zero-shot setting, Google Gemini 2.5 flash showed the highest accuracy at 73.8% (95% CI: 71.0–76.4%), with the highest specificity (88.9%, 95% CI: 84.7–92.2%) and PPV (94.0%, 95% CI: 91.9–95.6%). Google Gemini 2.5 pro achieved the highest sensitivity at 90.9% (95% CI: 88.7–92.9%) and F1-score at 82.4% (95% CI: 80.7–83.9%), although its specificity was low (23.3%, 95% CI: 18.6–28.6%). Conversely, Google Gemini 2.0 flash performed poorly across metrics, exhibiting the lowest accuracy (26.9%, 95% CI: 24.2–29.7%), sensitivity (11.4%, 95% CI: 9.3–13.9%), and PPV (46.8%, 95% CI: 40.5–53.1%). The OpenAI GPT-4.1 model demonstrated high accuracy (70.6%, 95% CI: 67.7–73.3%) and sensitivity (81.3%, 95% CI: 78.4–84.0%), balanced with a moderate PPV of 78.6% (95% CI: 76.7–80.3%). DeepSeek models exhibited intermediate accuracy, with DeepSeek V3 achieving an accuracy of 62.7% (95% CI: 59.7–65.6%) and DeepSeek R1 achieving 48.4% (95% CI: 45.4–51.5%). The corresponding binary confusion matrices for all evaluated models under the zero-shot condition are presented in Fig. 1 and detailed three-class matrices are provided in Supplementary Fig. 1.

**Table 3:** The performance metrics of chat model and reasoning model from OpenAI, Google and DeepSeek for triage in emergency department based on real-world conversation with Zero-Shot prompting

	GPT-4o	GPT-4.1	Gemini 2.0 flash	Gemini 2.5 flash	Gemini 2.5 pro	DeepSeek V3	DeepSeek R1
Accuracy (%) (95% CI)	56.3% (53.2%−59.3%)	70.6% (67.7%−73.3%)	26.9% (24.2%−29.7%))	73.8% (71.0%−76.4%)	72.0% (69.2%−74.7%)	62.7% (59.7%−65.6%)	48.4% (45.4%−51.5%)
Sensitivity (95% CI)	0.501 (0.464 − 0.537)	0.813 (0.784 − 0.840)	0.114 (0.093 − 0.139)	0.679 (0.645 − 0.712)	0.909 (0.887 − 0.929)	0.645 (0.610 − 0.679)	0.392 (0.357 − 0.427)
Specificity (95% CI)	0.723 (0.668 − 0.773)	0.429 (0.372 − 0.488)	0.666 (0.609 − 0.719)	0.889 (0.847 − 0.922)	0.233 (0.186 − 0.286)	0.581 (0.522 − 0.638)	0.723 (0.668 − 0.773)
PPV (95% CI)	0.823 (0.792 − 0.850)	0.786 (0.767 − 0.803)	0.468 (0.405 − 0.531)	0.940 (0.919 − 0.956)	0.753 (0.740 − 0.765)	0.798 (0.774 − 0.821)	0.784 (0.748 − 0.817)
NPV (95% CI)	0.360 (0.338 − 0.384)	0.472 (0.423 − 0.522)	0.226 (0.212 − 0.241)	0.519 (0.491 − 0.546)	0.500 (0.424 − 0.576)	0.389 (0.357 − 0.422)	0.316 (0.297 − 0.336)
F1-Score (95% CI)	0.623 (0.585 − 0.658)	0.799 (0.775 − 0.821)	0.183 (0.151 − 0.220)	0.788 (0.758 − 0.816)	0.824 (0.807 − 0.839)	0.713 (0.682 − 0.741)	0.523 (0.483 − 0.561)
Response Time (per case)	1.75s	1.79s	0.35s	2.34s	16.76s	1.49s	35.77s

PPV, Positive Predictive Value; NPV, Negative Predictive Value

A Cochran’s Q test was performed among the three models with accuracy greater than 70% in the zero-shot condition—OpenAI GPT-4.1, Google Gemini 2.5 flash, and Gemini 2.5 pro—and revealed statistically significant differences (*p* < 0.001). As a post-hoc analysis, paired McNemar tests with Bonferroni correction were conducted for each pair, and all comparisons remained statistically significant (GPT-4.1 vs. Gemini 2.5 flash, *p* < 0.0001, Difference − 22.52%, 95% CI: -25.58%–-19.46%; GPT-4.1 vs. Gemini 2.5 pro, *p* < 0.0001, Difference 12.39%, 95% CI: 10.03%-14.76%; Gemini 2.5 flash vs. Gemini 2.5 pro, *p* < 0.0001, Difference 34.91%, 95% CI: 31.92–37.90%). These differences show that there are statistically significant differences in the sensitivity and specificity of the three models.

Response times per case varied significantly among models. Google Gemini 2.0 flash provided the fastest responses averaging 0.35 s per case, while DeepSeek R1 exhibited the slowest average response time at 35.77 s per case. OpenAI GPT-4o and GPT-4.1 recorded average response times of 1.75 s and 1.79 s per case, respectively.

The cost of using the APIs of the LLMs used is presented in Supplementary Table 6. OpenAI O3 had the highest cost, totaling $5.13. Across all models, the cost per triage case ranged from $0.0001 to $0.005.

### Model performance with few-shot prompting

Under the few-shot prompting condition, the performance of all models improved compared with the zero-shot baseline. As summarized in Table 4, Gemini 2.5 flash achieved the highest overall accuracy at 73.5% (95% CI: 71.0–76.4%) and an F1-score of 0.829 (95% CI: 0.810–0.846). Gemini 2.5 pro demonstrated comparable results (accuracy 73.0%, F1-score 0.819), while DeepSeek V3 achieved an accuracy of 73.4% with an F1-score of 0.820, placing these three models as the top performers. In contrast, Gemini 2.0 flash and GPT-4o showed relatively lower performance, although still higher than in the zero-shot setting.

**Table 4:** The performance metrics of chat model and reasoning model from OpenAI, Google and DeepSeek for triage in emergency department based on real-world conversation with Few-Shot prompting

	GPT-4o	GPT-4.1	Gemini 2.0 flash	Gemini 2.5 flash	Gemini 2.5 pro	DeepSeek V3	DeepSeek R1
Accuracy (%) (95% CI)	60.4% (57.3%−63.3%)	68.4% (65.5%−71.2%)	61.5% (58.5%−64.4%))	73.5% (71.0%−76.4%)	73.0% (70.2%−75.7%)	73.4% (70.6%−76.1%)	60.7% (57.7%−63.7%)
Sensitivity (95% CI)	0.541 (0.505 − 0.577)	0.724 (0.691 − 0.756)	0.556 (0.520 − 0.592)	0.892 (0.868 − 0.913)	0.850 (0.823 − 0.875)	0.842 (0.814 − 0.868)	0.606 (0.570 − 0.641)
Specificity (95% CI)	0.764 (0.711 − 0.811)	0.581 (0.523 − 0.638)	0.767 (0.714 − 0.814)	0.331 (0.278 − 0.388)	0.422 (0.365 − 0.481)	0.456 (0.398 − 0.515)	0.611 (0.553 − 0.667)
PPV (95% CI)	0.855 (0.826 − 0.879)	0.816 (0.794 − 0.837)	0.860 (0.832 − 0.884)	0.774 (0.759 − 0.789)	0.791 (0.773 − 0.807)	0.799 (0.781 − 0.816)	0.800 (0.775 − 0.824)
NPV (95% CI)	0.393 (0.369 − 0.417)	0.450 (0.413 − 0.488)	0.402 (0.378 − 0.426)	0.544 (0.479 − 0.608)	0.523 (0.469 − 0.576)	0.529 (0.478 − 0.580)	0.376 (0.347 − 0.406)
F1-Score (95% CI)	0.663 (0.627 − 0.697)	0.767 (0.738 − 0.794)	0.675 (0.640 − 0.709)	0.829 (0.810 − 0.846)	0.819 (0.797 − 0.840)	0.820 (0.797 − 0.841)	0.690 (0.657 − 0.721)
Response Time (per case)	5.52s	5.55s	0.66s	3.44s	13.38s	3.81s	28.25s

PPV, Positive Predictive Value; NPV, Negative Predictive Value

**Fig. 2:**
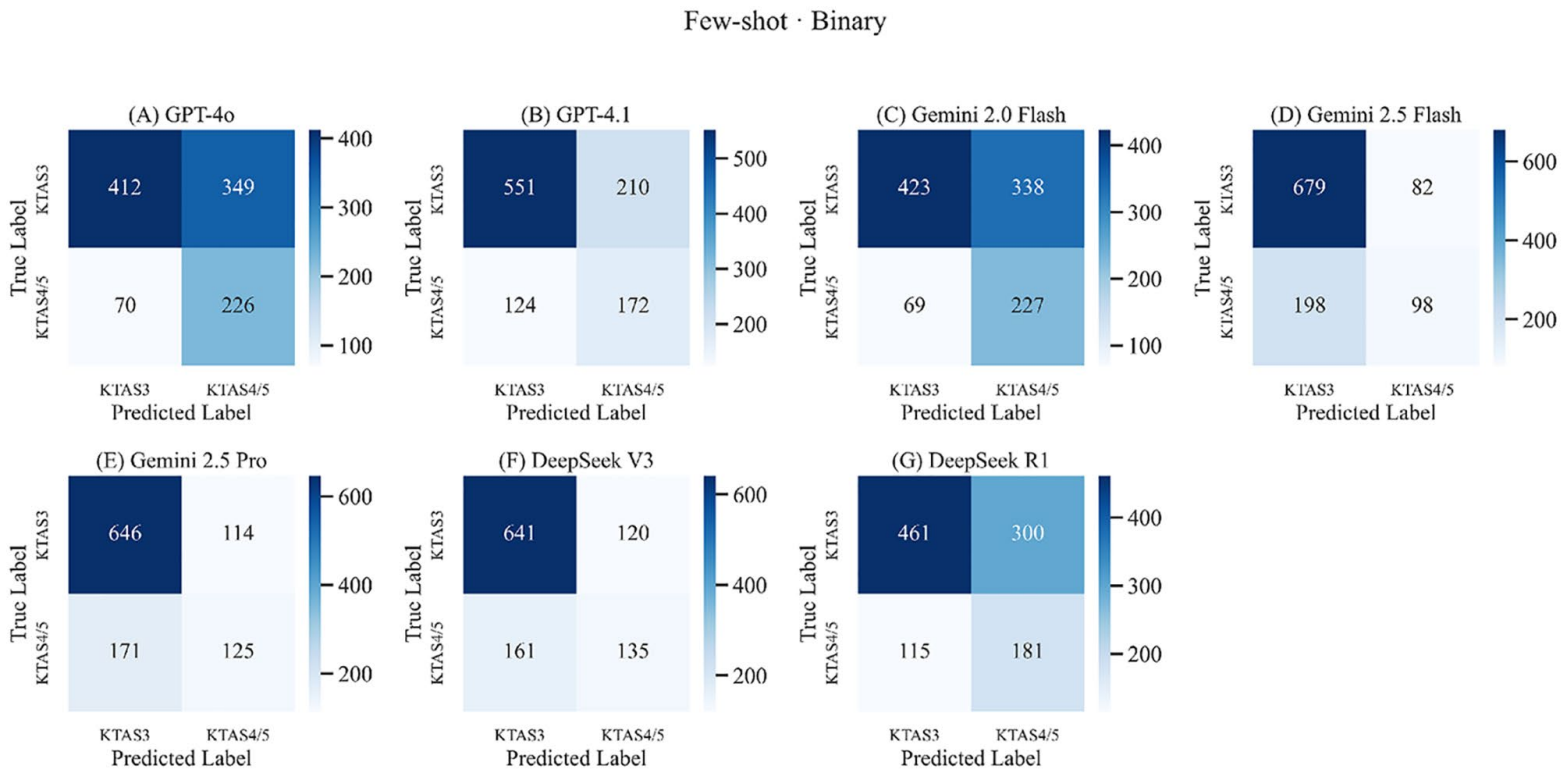
Binary confusion matrices for all evaluated models under the Few-Shot condition. (**A**) GPT-4o (**B**) GPT-4.1 (**C**) Gemini 2.0 Flash (**D**) Gemini 2.5 Flash (**E**) Gemini 2.5 Pro (**F**) DeepSeek V3 (**G**) DeepSeek R1. Each heatmap illustrates classification outcomes for urgent (KTAS 3) vs. non-urgent (KTAS 4–5) patients

Compared with zero-shot prompting, the few-shot strategy consistently improved sensitivity and F1-scores across nearly all models. Notably, Gemini 2.0 flash, which had previously shown very low accuracy and sensitivity, demonstrated marked gains in performance under the few-shot conditions. Accuracy improved or remained stable except for GPT-4.1. These findings suggest that few-shot prompting reduced the risk of under-triage and improved the balance between sensitivity and specificity. The corresponding binary confusion matrices for all models under the few-shot condition are presented in Fig. 2 and detailed three-class matrices are provided in Supplementary Fig. 2.

Within the few-shot prompting condition, accuracy differed significantly across the three top-performing models—Gemini 2.5 flash, Gemini 2.5 pro, and DeepSeek V3—based on paired case-level accuracy (Cochran’s Q test, *p* < 0.0001). Post-hoc pairwise McNemar tests showed that Gemini 2.5 flash differed significantly from both Gemini 2.5 pro (*p* < 0.0001, Difference − 5.58%, 95% CI: -7.77%–-3.39%) and DeepSeek V3 (*p* < 0.0001, Difference − 7.10%, 95% CI: -9.32%–-4.87%), whereas the difference between Gemini 2.5 pro and DeepSeek V3 was not statistically significant (*p* = 0.2205, Difference − 1.51%, 95% CI: -3.78–0.76%).

Regarding response times, Gemini 2.0 flash remained the fastest model at an average of 0.66 s per case, whereas DeepSeek R1 remained the slowest at 28.25 s per case When compared with the zero-shot condition, response times generally increased in non-reasoning models, reflecting the additional processing required for contextual instructions, while reasoning models such as GPT-4.1 and Gemini 2.5 pro demonstrated shorter response times, suggesting that structured prompting enhanced efficiency.

The costs of using the models under the few-shot conditions are detailed in Supplementary Table 7. Compared with the zero-shot condition, costs increased across all models, with the rise most pronounced in reasoning-capable models.

Because O3 uses fixed generation parameters (temperature = 1.0) that could not be aligned with the other models, it was excluded from head-to-head statistical comparisons. For completeness, its performance metrics are reported in Supplementary Table 8. Its binary and three-class confusion matrices are presented in Supplementary Fig. 3.

### Failure analysis

In the zero-shot condition, a total of 62 discordant cases were identified, consisting of 26 cases of over-triage and 36 cases of under-triage where all three top-performing models (GPT-4.1, Gemini 2.5 flash, and Gemini 2.5 pro) produced identical results that did not match the nurse-assigned KTAS levels.

Among the 26 cases of over-triage, one was attributable to a nurse error (a patient presenting with jaundice), while the remaining 25 reflected misclassification by the models. The most frequent pattern occurred in patients with minor injuries, where the models tended to over-triage whenever pain or bleeding was mentioned during the conversation (13 cases). Head trauma was particularly prone to over-triage, as the models classified patients as urgent regardless of the actual mechanism or severity of injury. A similar trend was observed in non-trauma cases involving bleeding, such as cystitis with hematuria or epistaxis with minor bleeding. These findings suggest that the models may disproportionately weight specific keywords such as “pain,” “bleeding,” or “head injury,” leading to systematic overestimation of acuity.

Among the 36 under-triage cases, three were attributable to nurse errors (a patient with a fish bone lodged in the throat, cystitis, and a finger laceration), in which the consensus physician review and the models appeared to provide more appropriate classifications. The remaining 33 cases reflected model misclassification. Twelve involved patients referred from outpatient clinics or other hospitals Because such information was not available in the conversation transcripts, the models classified these patients at lower acuity. An additional example of such a misclassification is provided in Supplementary Table 9, illustrating an under-triage case in which a KTAS level 3 patient referred from the outpatient clinic was incorrectly classified as level 4. Other under-triage cases included patients with dizziness, multiple trauma patients, patients with severe sore throat and difficulty swallowing, patients with abdominal pain, and patients with dyspnea These findings highlight the limitations of relying solely on conversational data, as missing contextual information and patient-reported expressions that downplay severity often resulted in systematic under-triage.

## Discussion

This study adds a novel contribution to research on LLMs for emergency department triage. Unlike previous studies that rely primarily on scenario-based or summarized clinical vignettes, this study used real-world, unsummarized clinical conversations from ED interactions [11–14]. It also provides a comprehensive comparison of several commercially available LLMs, including OpenAI, Google, and DeepSeek, providing a practical reference for model selection in clinical settings. Despite the absence of objective clinical data such as vital signs, the LLMs accurately inferred patient severity using only symptoms and conversational context.

Several models demonstrated reliable performance even with basic zero-shot prompting, while others showed substantial improvements when few-shot prompting was applied. These findings highlight the clinical utility of advanced LLMs for triage support, as their accuracy and consistency improved with relatively simple adjustments in prompt design. Importantly, this study was limited to non-critical patients (KTAS levels 3–5), focusing on the challenging task of distinguishing patients who appear non-urgent but may in fact require timely intervention. Within this scope, LLMs are not intended to replace human triage, particularly for critically ill patients (KTAS 1–2), but rather to serve as decision-support tools that can complement clinical judgment. From a practical standpoint, such tools may enhance patient safety and resource allocation, especially by improving the identification of KTAS 3 patients who are at risk of deterioration if under-triaged.

In this study, we evaluated the performance of LLMs using only unsummarized real-world conversations. Previous studies evaluated the performance of LLMs in triage using structured clinical scenarios or vignettes with summarized vital signs, comorbidities, and clinical information [11–14]. In our study, LLMs showed performance comparable to the best model in previous studies, which reported accuracies ranging from 60 to 70% [11–13]. A recent study by Pasli et al. reported high accuracy in patient triage using GPT-4o [15]. However, their approach required personnel to manually input the structured information such as chief complaint, comorbidities, and vital signs into the model. In contrast, our study directly employed LLMs to analyze and classify unstructured conversations between triage nurses and patients without additional personnel or structured inputs. This methodology significantly enhances practical applicability.

By integrating automatic speech recognition, our approach could be readily implemented in clinical practice, streamlining workflows and reducing staffing needs [18]. Recent evaluations of ASR performance have shown that Whisper achieves a character error rate of approximately 5.2% on ED dialogue datasets, demonstrating high transcription accuracy [16]. In addition, optimized streaming implementations of Whisper reported average latencies of about 3.3 s [19]. These latencies are broadly compatible with real-time triage requirements. Taken together, these findings suggest that ASR can serve as a viable component of live triage pipelines, although further optimization remains essential to ensure seamless clinical integration.

Our results showed notable performance variability among different models. Specifically, Gemini 2.5 flash, 2.5 pro and GPT-4.1 displayed high accuracy (73.8%, 72.0% and 70.6% respectively), reinforcing the potential clinical utility of advanced LLMs. Nonetheless, significant differences among versions within the same platform highlight the importance of careful model validation prior to clinical implementation. With few-shot prompting, most models demonstrated further improvements, particularly in F1-scores, even in cases where overall accuracy changed only modestly. For example, Gemini 2.0 flash, which performed poorly with zero-shot prompting (26.9% accuracy), showed marked gains under few-shot conditions, while Gemini 2.5 pro achieved higher F1-scores despite minimal changes in accuracy.

A key finding pertains to the sensitivity-specificity trade-off observed, particularly in the Gemini 2.5 pro model. With exceptionally high sensitivity (90.9%), this model effectively identified nearly all urgent cases, a critical advantage in emergency medicine, where under-triage carries substantial risk. However, the model’s low specificity (23.3%) indicates frequent over-triage of non-urgent patients. Thus, the clinical deployment of LLMs should aim to enhance specificity without compromising sensitivity significantly. Few-shot prompting mitigated some of these trade-offs by improving balance in overall classification performance. Therefore, optimizing prompt strategies to refine model behavior is important for clinical implementation.

Response time emerged as an essential consideration. Although Gemini 2.0 flash was the fastest model (0.35 s per case), its poor accuracy (26.9%) limits clinical relevance. Gemini 2.5 pro showed high accuracy but required substantially longer response times (16.76 s per case), potentially hindering real-time clinical application. GPT-4.1 achieved a balanced performance, showing relatively high accuracy alongside acceptable response times (1.79 s per case). With few-shot prompting, reasoning-oriented models such as DeepSeek R1 showed reduced latency, while non-reasoning models exhibited somewhat slower responses, reflecting trade-offs introduced by more complex prompting. Therefore, clinical adoption of LLMs necessitates a careful balance between speed and accuracy to meet practical ED needs effectively.

Interestingly, reasoning-oriented models such as OpenAI O3 and DeepSeek R1 needed more time and underperformed relative to simpler counterparts. This unexpected outcome suggested that increased model complexity may introduce unnecessary challenges for straightforward clinical tasks [20]. Because both speed and accuracy are important in emergency room triage, future studies should clarify the conditions under which complex AI reasoning capabilities offer meaningful clinical advantages.

The failure analysis provided insights beyond overall performance metrics. Over-triage frequently occurred when models encountered keywords such as bleeding, trauma, or pain. These cues often led to urgent classifications, even in minor conditions. Under-triage was common when critical context was missing. This was particularly evident in referred patients, where the reason for transfer was not clearly expressed in the dialogue. Other examples were due to missing contextual information and patient-reported expressions. In minor cases, nurse errors contributed to discrepancies. These patterns suggest that LLMs may over-rely on certain cues and underperform when contextual information is absent. Therefore, LLMs should be used as decision-support tools than as replacements for humans.

Another important aspect of this study is its direct utilization of Korean-language data without translation. This highlights the robust performance of certain advanced LLMs, such as GPT-4.1 and Gemini 2.5 pro, in non-English contexts. Typically, LLM performance is reported to decline significantly when handling languages other than English due to limitations in tokenizer effectiveness or inadequate representation in training datasets [21]. Clinically, this finding is encouraging, as it demonstrates the feasibility of effectively integrating LLM-based triage tools in emergency settings across diverse linguistic and cultural contexts. It also extends the utility of these advanced models beyond English-speaking environments.

Rapid and accurate triage requires extensive training and experience among ED personnel. In contrast, the LLMs tested in this study demonstrated reasonable performance without any specific instruction, real-world experience in KTAS, or triage practice. This finding suggests that dedicated triage-specific LLMs could be developed to achieve even higher accuracy with sufficient case data and appropriate feedback. Such models could provide real-time support to less experienced clinicians, potentially improving triage efficiency and safety, especially during periods of overcrowding. In addition, the LLMs achieved these results solely from dialogue, without access to vital signs, a core component of traditional triage. This raises the possibility of using conversational LLMs in prehospital telephone triage (such as 911), or in situations where in-person assessment is limited, such as during pandemic-related lockdowns (e.g. Coronavirus disease 2019) or for remote/telemedicine care. Also, integrating LLMs with other AI-based tools may further enhance their capabilities, paving the way for advanced multimodal decision support systems in emergency care.

## Limitations

This study has several limitations. First, triage classifications performed by nurses were used as the gold standard. This potentially introduces bias due to inherent inaccuracies in human triage decisions. For reference, one study reported a 14.7% error rate when nurses triage KTAS [22]. However, in our study, an additional validation of 50 randomly selected cases demonstrated high agreement between nurse-assigned and physician-assigned classifications, supporting the overall reliability of the gold standard used. Second, the design of prompts could significantly affect performance outcomes, particularly in few-shot prompting, where the choice of illustrative examples may alter the reported accuracies. Third, the scope of this study was limited to non-critical patients (KTAS levels 3–5). Patients with KTAS 1–2 were excluded for ethical and practical reasons, and KTAS 4 and 5 were merged into a single “non-urgent” category due to sample size and imbalance. Therefore, the proposed approach should not be generalized to critically ill patients or to the entire triage process. Nevertheless, improving the identification of KTAS 3 patients within the KTAS 3–5 range remains clinically meaningful, as these patients may deteriorate if under-triaged. Fourth, although prior work has demonstrated that ASR systems such as Whisper can achieve high accuracy and near real-time latency, our study did not directly integrate ASR. Future research should evaluate its effect on triage performance in real-world workflows. In addition, the deployment of commercial LLM APIs in clinical practice inherently raises privacy concerns, since patient data may be transmitted to external servers. While our study avoided this issue by using fully de-identified data, real-world implementation will likely require on-premise or institutionally hosted LLMs to ensure compliance with privacy regulations. Moreover, the question of liability for AI-related errors remains unresolved, underscoring that such systems should only be used as decision-support tools with human oversight and within clear accountability frameworks. Fifth, this study was conducted entirely in Korean, so the results cannot be directly generalized to other languages or cultural contexts. However, the findings are encouraging for the use of LLM in non-English speaking situations. Finally, while the accuracy of LLM using only conversational data is encouraging, it is important to recognize that the incorporation of objective clinical information, such as vital signs, is still essential to ensure patient safety in real-world triage. In addition, artificial intelligence should be emphasized as a supportive tool, not a replacement for clinical judgment or medical professionals.

## Conclusion

This study showed that LLMs can accurately triage emergency patients using only real-world clinical conversations. Several models demonstrated both high sensitivity and practical response times. These findings support the potential for LLM-based triage tools to improve emergency care in diverse clinical settings.

## Supplementary Information

Below is the link to the electronic supplementary material.

Supplementary Material 1

## Data Availability

The data used in this study is publicly available on the AI Hub, administered by the National Information Society Agency in South Korea.
