# Supplementary material for: Performance of ChatGPT, Gemini and DeepSeek for non-critical triage support using real-world conversations in emergency department
Source: BMC Emerg Med. 2025 Sep 1;25:176. doi: 10.1186/s12873-025-01337-2 (PMC12403343; doi:10.1186/s12873-025-01337-2)

**Supplementary Table 1.** The version of the application programming interface (API) of the large language model (LLM) used.

| **Model** | **API Version** | **Last Update** |
| --- | --- | --- |
| OpenAI GPT-4o | GPT-4o-2024-08-06 | August 2024 |
| OpenAI GPT-4.1 | GPT-4.1-2025-04-14 | April 2025 |
| OpenAI O3 | O3-2025-04-16 | April 2025 |
| Google Gemini 2.0 flash | Gemini-2.0-flash | February 2025 |
| Google Gemini 2.5 flash | Gemini-2.5-flash | June 2025 |
| Google Gemini 2.5 pro | Gemini-2.5-pro | June 2025 |
| DeepSeek V3 | DeepSeek-V3-0324 | March 2025 |
| DeepSeek R1 | DeepSeek-R1-0528 | May 2025 |

**Supplementary Table 2.** The Zero-Shot system prompt entered in the application programming interface (API) of a large-scale language model (LLM)

| You are a triage assistant trained to classify patients based on the Korean Triage and Acuity Scale (KTAS).  Your task is to read a dialogue between a healthcare provider and a patient and classify the patient's acuity level.  Important constraints:  1. You MUST choose only one of the following KTAS levels: **3, 4, or 5**.  2. DO NOT choose KTAS level 1 or 2 under any circumstances.  3. Your response MUST be a single number: `3`, `4`, or `5`. Do not include any explanation or extra text.  Below is the transcript of the patient-provider conversation: |
| --- |

**Supplementary Table 3.** The Few-Shot system prompt entered in the application programming interface (API) of a large-scale language model (LLM).

| You are a triage assistant trained to classify patients based on the Korean Triage and Acuity Scale (KTAS).  Your task is to read a dialogue between a healthcare provider and a patient and classify the patient's acuity level.  If you don't have enough information for triage (e.g., vital signs), infer severity from the context of the conversation.  If you're still unsure about your triage classification, try to categorize it in a way that avoids under-triage as much as possible.  Important constraints:  1. You MUST choose only one of the following KTAS levels: **3, 4, or 5**.  2. DO NOT choose KTAS level 1 or 2 under any circumstances.  3. Your response MUST be a single number: `3`, `4`, or `5`. Do not include any explanation or extra text.  4. DO NOT summarize the current situation. Answer only the conclusion as '3' or '4' or '5'.  Below is the transcript of the patient-provider conversation: |
| --- |

Examples for Few-Shot were provided separately through the API, and the examples used are presented in Table 1 and the supplement table.

**Supplementary Table 4.** An example of a clinical conversation in the emergency department used in the study (KTAS level 4). The original conversation is in Korean, and the examples have been translated into English for better understanding.

| **No.** | **Speaker** | **Conversations** |
| --- | --- | --- |
| 1 | Triage nurse | This way please. Take a sit. What makes you come here today? |
| 2 | Guardian | He hurt his right arm. |
| 3 | Triage nurse | Where does his right arm hurt? Is it his shoulder? Is it his elbow? |
| 4 | Guardian | It's his shoulder. |
| 5 | Triage nurse | Is it his right shoulder? |
| 6 | Guardian | Yes |
| 7 | Triage nurse | How did he get injured? |
| 8 | Guardian | He slipped down, falling to the floor. |
| 9 | Triage nurse | When did that happen? |
| 10 | Patient | Just a moment ago. |
| 11 | Triage nurse | About ten minutes ago? |
| 12 | Patient | From the ladder. |
| 13 | Triage nurse | From the ladder? |
| 14 | Triage nurse | Are there any other areas that hurt besides your shoulder? |
| 15 | Patient | Only the right shoulder. |
| 16 | Triage nurse | Only your shoulder hurts? |
| 17 | Triage nurse | Did you hit your head? |
| 18 | Triage nurse | What time did it happen? |
| 19 | Patient | About thirty minutes ago. |
| 20 | Triage nurse | Thirty minutes ago. |
| 21 | Patient | Yes, I think so. |
| 22 | Triage nurse | How does it hurt? Does it hurt like a sharp pain or a dull pain? |
| 23 | Patient | It feels like tingling. |
| 24 | Triage nurse | Does it hurt when I touch it? |
| 25 | Patient | Yes. |
| 26 | Triage nurse | How is your sensation? |
| 27 | Patient | I have sensation. |
| … |  |  |

**Supplementary Table 5.** An example of a clinical conversation in the emergency department used in the study (KTAS level 5). The original conversation is in Korean, and the examples have been translated into English for better understanding.

| **No.** | **Speaker** | **Conversations** |
| --- | --- | --- |
| 1 | Triage nurse | Please come this way. |
| 2 | Triage nurse | What brought you to the emergency room? |
| 3 | Patient | I came to have my wound dressed. |
| 4 | Triage nurse | Did you come to have your wound dressed today? |
| 5 | Patient | Yes. |
| 6 | Triage nurse | Let's check your blood pressure first. |
| 7 | Triage nurse | Is the wound only on your forehead? |
| 8 | Patient | Yes. |
| 9 | Triage nurse | It's only on your forehead. |
| 10 | Patient | Yes. |
| 11 | Triage nurse | Just on your forehead, right? |
| 12 | Patient | Yes. |
| 13 | Patient | The bruise is getting lower. |
| 14 | Triage nurse | Lower? |
| 15 | Patient | Yes. |
| 16 | Triage nurse | It might get darker in color. That's possible. |
| 17 | Triage nurse | We'll check the wound and your blood pressure first, then go inside. |
| 18 | Triage nurse | Please sit on the chair on the right. |

**Supplementary Table 6.** The cost of using the application programming interface (API) of the large-scale language model (LLM) used with Zero-Shot prompting.

| **Model** | **Total cost** | **Cost per case** |
| --- | --- | --- |
| OpenAI GPT-4o | $ 2.40 | $ 0.0023 |
| OpenAI GPT-4.1 | $ 1.99 | $ 0.0019 |
| OpenAI O3 | $ 5.13 | $ 0.0049 |
| Google Gemini 2.0 flash | $ 0.11 | $ 0.0001 |
| Google Gemini 2.5 flash | $ 2.67 | $ 0.0025 |
| Google Gemini 2.5 pro | $ 1.13 | $ 0.0011 |
| DeepSeek V3 | $ 0.26 | $ 0.0002 |
| DeepSeek R1 | $ 1.62 | $ 0.0015 |

* Google Gemini was paid in Korean won, which was calculated as $1 = ₩ 1,360.

**Supplementary Table 7.** The cost of using the application programming interface (API) of the large-scale language model (LLM) used with Few-Shot prompting.

| **Model** | **Total cost** | **Cost per case** |
| --- | --- | --- |
| OpenAI GPT-4o | $ 4.89 | $ 0.0046 |
| OpenAI GPT-4.1 | $ 2.94 | $ 0.0028 |
| OpenAI O3 | $ 5.73 | $ 0.0054 |
| Google Gemini 2.0 flash | $ 0.32 | $ 0.0003 |
| Google Gemini 2.5 flash | $ 2.05 | $ 0.0019 |
| Google Gemini 2.5 pro | $ 18.06 | $ 0.0171 |
| DeepSeek V3 | $ 0.42 | $ 0.0004 |
| DeepSeek R1 | $ 1.07 | $ 0.0010 |

* Google Gemini was paid in Korean won, which was calculated as $1 = ₩ 1,360.

**Supplementary Table 8.** The performance metrics of OpenAI’s O3 for triage in emergency department based on real-world conversation with Zero-Shot prompting.

|  | O3 with Zero-Shot Prompt | O3 with Few-Shot Prompt |
| --- | --- | --- |
| Accuracy (%)  (95% CI) | 66.2%  (63.3%−69.1%) | 71.0%  (68.1%−73.7%) |
| Sensitivity  (95% CI) | 0.716  (0.784−0.840) | 0.778  (0.747−0.807) |
| Specificity  (95% CI) | 0.524  (0.465−0.582) | 0.534  (0.475−0.592) |
| PPV  (95% CI) | 0.794  (0.773−0.815) | 0.811  (0.791−0.830) |
| NPV  (95% CI) | 0.418  (0.380−0.456) | 0.483  (0.441−0.526) |
| F1-Score  (95% CI) | 0.753  (0.725−0.780) | 0.800  (0.768−0.818) |
| Response Time  (per case) | 9.48s | 6.14s |

PPV, Positive Predictive Value; NPV, Negative Predictive Value

**Supplementary Table 9.** Clinical conversation example of an under-triage failure case where a KTAS level 3 patient was misclassified as level 4.

| **No.** | **Speaker** | **Conversations** |
| --- | --- | --- |
| 1 | Triage nurse | What brings you to the emergency room today? |
| 2 | Patient | Yesterday, on the 31st, I was told I would be admitted here. |
| 3 | Patient | But they said my kidney results were too high and told me to come in quickly. |
| 4 | Triage nurse | Kidney? |
| 5 | Patient | Kidney. |
| 6 | Patient | They told me to come right away. |
| 7 | Triage nurse | Did the test results come out today? |
| 8 | Patient | Around 2 p.m. they called me. |
| 9 | Triage nurse | From our hospital? |
| 10 | Patient | Yes, they told me to come in quickly. |
| 11 | Triage nurse | So, they said your kidney values weren’t good? |
| 12 | Triage nurse | What exactly did the hospital tell you? |
| 13 | Guardian | Wasn’t it calcium that was high? |
| 14 | Patient | They said my potassium was high. |
| 15 | Triage nurse | Potassium? |
| 16 | Patient | Yes, I think so. |
| 17 | Triage nurse | Other than the lab results, do you have any symptoms? |
| 18 | Patient | No. |
| 19 | Triage nurse | So, the only thing is that your potassium level was high? |
| 20 | Patient | Isn’t that written in your chart? |
| 21 | Triage nurse | I’m just trying to confirm what you were told. |
| 22 | Patient | I think that’s what they said on the phone. |
| 23 | Triage nurse | Do you have any medical conditions like hypertension or diabetes? |
| 24 | Patient | Conditions? I take medicine from neurology, nephrology, and endocrinology. |
| 25 | Triage nurse | For which conditions? |
| 26 | Guardian | Hypertension, diabetes, and he also had gout. |
| 27 | Patient | Hyperlipidemia. |
| 28 | Guardian | And kidney problems, so he’s on kidney medication. |
| … |  |  |

**Supplementary Figure 1.** Three-class confusion matrices (KTAS 3, 4, 5) for all evaluated models under Zero-Shot condition. (A) GPT-4o (B) GPT-4.1 (C) Gemini 2.0 Flash (D) Gemini 2.5 Flash (E) Gemini 2.5 Pro (F) DeepSeek
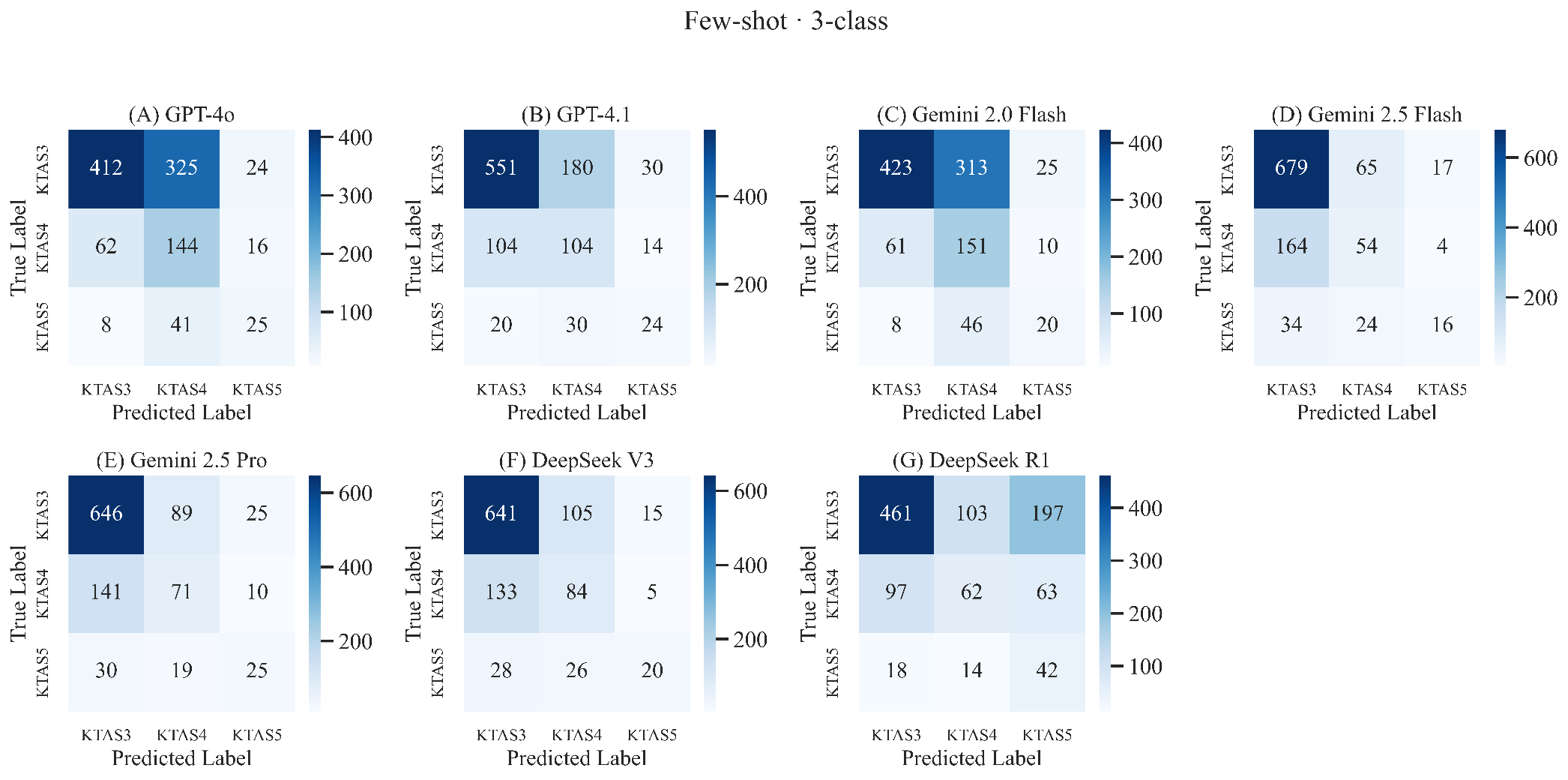
V3 (G) DeepSeek R1.

**Supplementary Figure 2.** Three-class confusion matrices (KTAS 3, 4, 5) for all evaluated models under Few-Shot condition. (A) GPT-4o (B) GPT-4.1 (C) Gemini 2.0 Flash (D) Gemini 2.5 Flash (E) Gemini 2.5 Pro (F) DeepSeek V3 (G) DeepSeek R1.
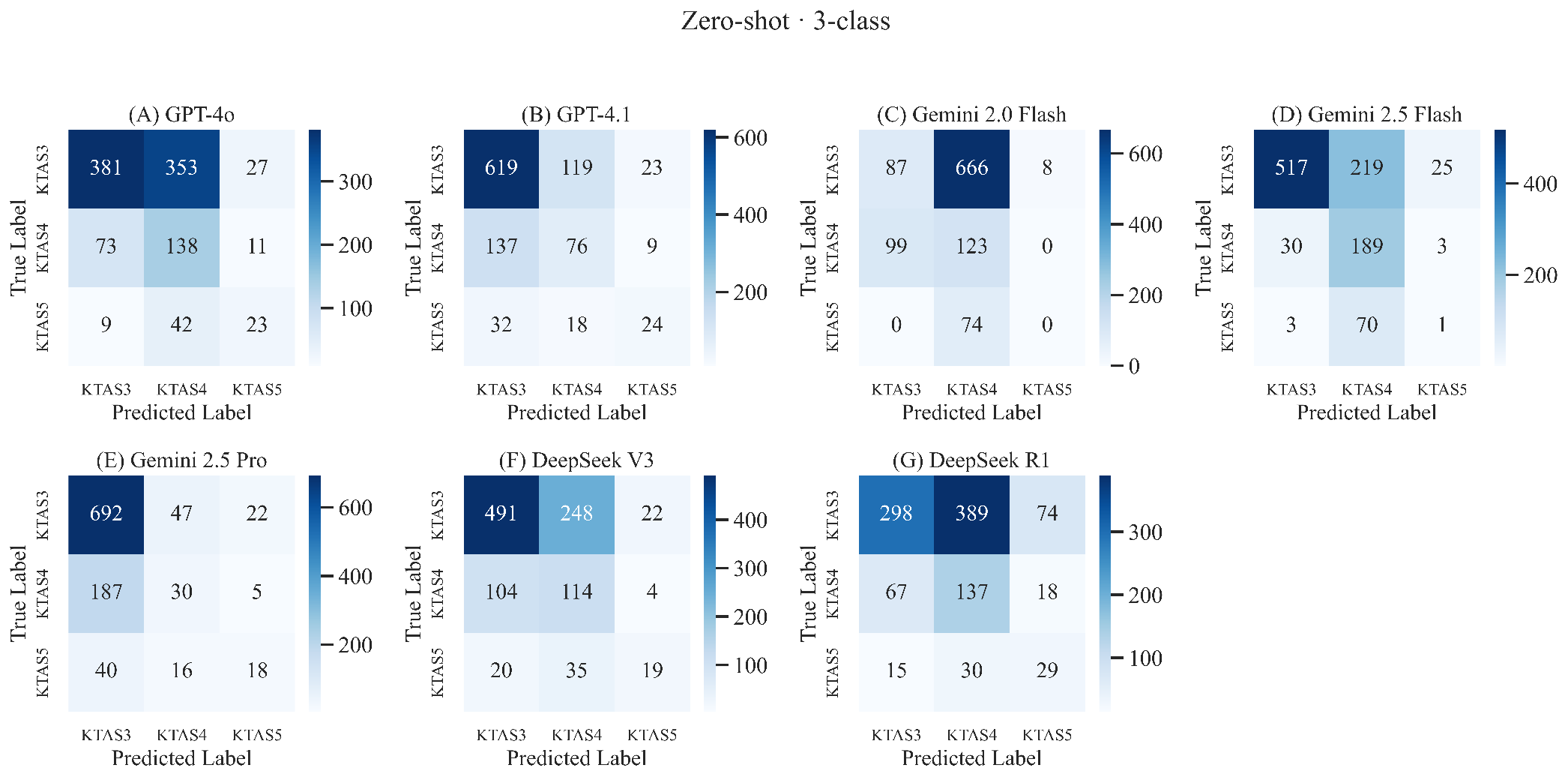


**Supplementary Figure 3.** Confusion matrices (binary and three-class) for the OpenAI O3 model. (A) Binary confusion matrix with Zero-Shot condition (B) Three-class confusion matrix with Zero-Shot condition (C) Binary confusion matrix with Few-Shot condition (D) Three-class confusion matrix with Few-Shot condition.


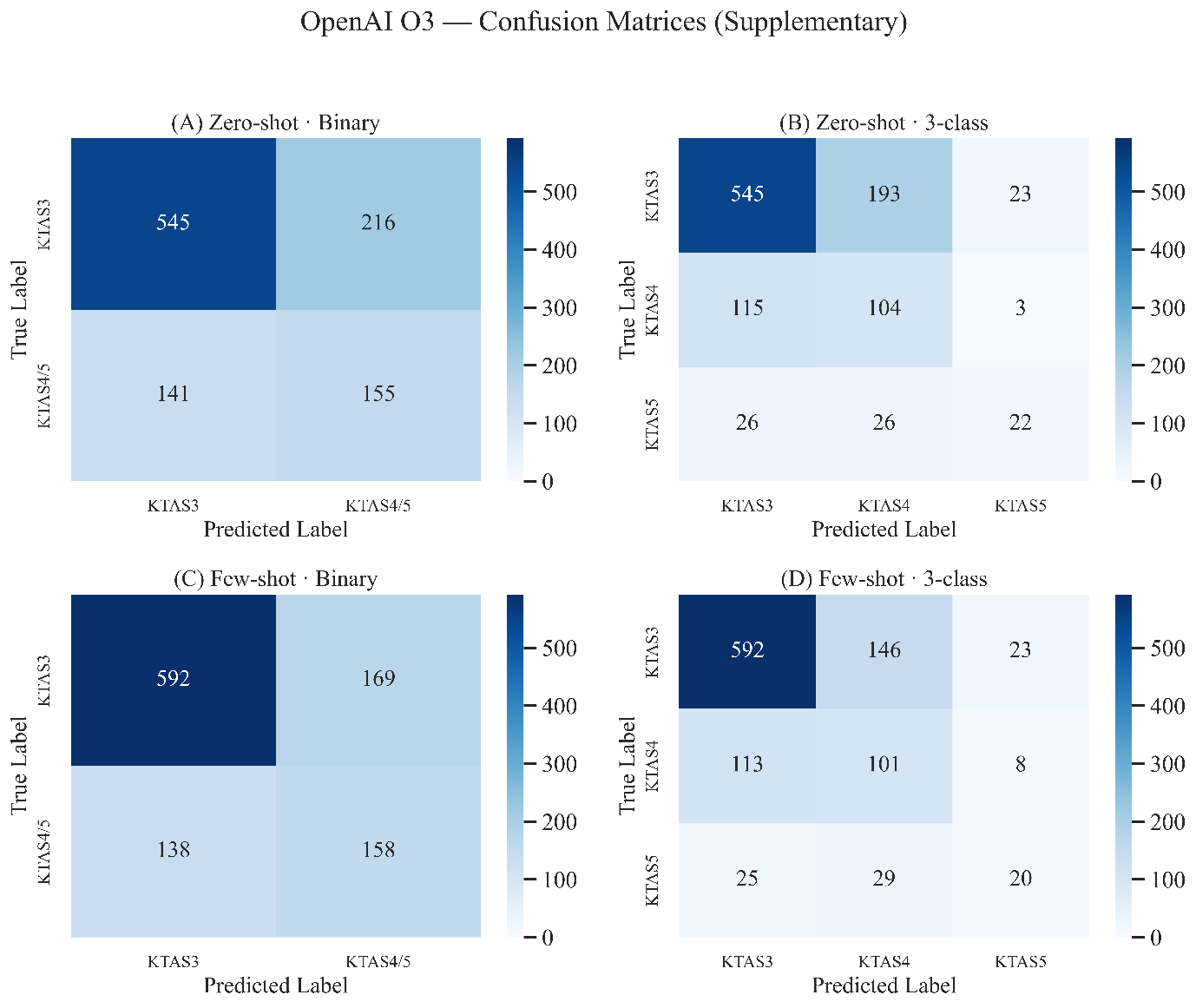

Supplement: Supplementary file 1 — Supplementary Material 1 [file 12873_2025_1337_MOESM1_ESM.docx]
